# The Effect of Visual Representation Style in Problem-Solving: A Perspective from Cognitive Processes

**DOI:** 10.1371/journal.pone.0080550

**Published:** 2013-11-15

**Authors:** Enkhbold Nyamsuren, Niels A. Taatgen

**Affiliations:** Department of Artificial Intelligence, University of Groningen, Groningen, Netherlands; University Medical Center Goettingen, Germany

## Abstract

Using results from a controlled experiment and simulations based on cognitive models, we show that visual presentation style can have a significant impact on performance in a complex problem-solving task. We compared subject performances in two isomorphic, but visually different, tasks based on a card game of SET. Although subjects used the same strategy in both tasks, the difference in presentation style resulted in radically different reaction times and significant deviations in scanpath patterns in the two tasks. Results from our study indicate that low-level subconscious visual processes, such as differential acuity in peripheral vision and low-level iconic memory, can have indirect, but significant effects on decision making during a problem-solving task. We have developed two ACT-R models that employ the same basic strategy but deal with different presentations styles. Our ACT-R models confirm that changes in low-level visual processes triggered by changes in presentation style can propagate to higher-level cognitive processes. Such a domino effect can significantly affect reaction times and eye movements, without affecting the overall strategy of problem solving.

## Introduction

More often than not, the study of problem solving is approached from the perspective of logical and rational thinking. In an early study, Weitzenfeld [1] defined the *isomorphic structure* of a task in terms of its elements and the relationship between those elements. Weitzenfeld further claimed that the isomorphic structure defines the strategy for accomplishing the task. However, Weitzenfeld ignored the significant impact presentation style can have on problem solving even when the isomorphic structure is preserved. Weitzenfeld took two games as an example of structure preservation: Tic-Tac-Toe and Number Scrabble. In Number Scrabble, players select in turn one of the remaining numbers from a pile that contains the numbers from 1 to 9. A player who is first to collect a triad of numbers adding up to 15 wins the game. Tic-Tac-Toe and Number Scrabble are valid isomorphic tasks. Isomorphism is obvious if numbers in Number Scrabble are arranged into a magic square where each column and row adds up to 15. Although it is highly likely that the two games require the same strategy, they are fundamentally different in terms of cognitive processes applied due to differences in visual presentation. While Number Scrabble requires top-down addition and subtraction, Tic-Tac-Toe requires more intuitive spatial reasoning [2]. Furthermore, such a difference in presentation styles may affect a player's performance independently of the strategy applied. For example, Michon [2] speculates that JAM, another game isomorphic to Tic-Tac-Toe, is easier to learn than Tic-Tac-Toe due to the fact that it has a different presentation.

**Figure 1:**
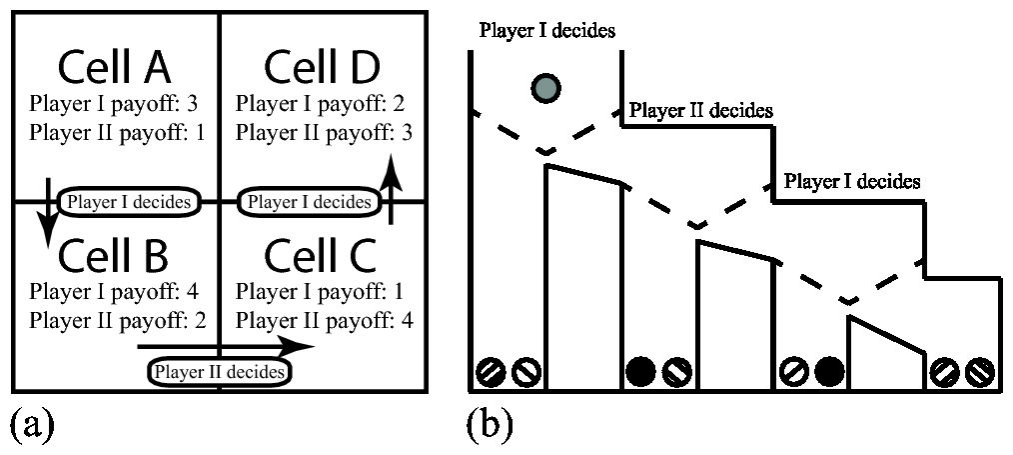
The Matrix game (a) used in [4] and its Marble Drop equivalent (b) described in [3].

In more recent work, Meijering, Van Maanen, Van Rijn and Verbrugge [3] showed that performance can differ significantly in two isomorphic tasks due to a change in visual presentation only. They did a comparative study of subjects' performances in Matrix and Marble Drop games. Hedden and Zhang [4] originally developed the Matrix game to study higher-order reasoning. Marble Drop is isomorphic to the Matrix game, albeit having a very different presentation style (Figure 1). In the Matrix game, each cell contains two separate payoffs for players. The game starts in cell A. Players make decisions in turns and can choose to either switch to a next cell or stay in a current cell. The game finishes when a player chooses to stay, or when cell D is reached. A player's goal is to finish the game in a cell with a maximum possible payoff. The Marble Drop game replaces numeric payoffs with color-graded marbles and cells with bins of decreasing height. Through manipulation of the trapdoors, a player has the choice to drop a marble to either the current bin or to the next set of trap doors controlled by the other player. Although the same chain of reasoning is required in both tasks, subjects showed superior reaction times and accuracy in the Marble Drop game.

This isomorphic structure may be appropriate in explaining players' strategies at a high level, but it is certainly not enough to explain the performance difference shown in [3]. So how does presentation style change the way humans approach a problem-solving task? After all, it is possible that a change in presentation style imposes a completely different strategy. However, in light of previous studies, it is an unlikely explanation. Alternatively, it can be the case that the overall strategy is the same, but specific actions within that strategy are performed in different ways depending on presentation styles. It is possible that the effects from those relatively small changes can accumulate and result in a significant difference in performance. 

There is evidence that individual steps within a strategy in the same task can be done differently, depending on a player's experience. For example, part of a common strategy in Tetris is to rotate and move a token to check where it fits best. This manipulation of tokens is done either physically or mentally, depending on a player's experience [5,6]. A similar effect is also observed in players playing Scrabble. Some players prefer to rearrange letters physically to check what valid words the letters can form [7]. Other players prefer to do the same step mentally. Experienced players who do mental manipulations generally perform better in both games. These examples show that the same actions in the same strategy can result in differences in performance if done in different ways.

In Tetris and Scrabble, we see a straightforward substitution of a physical process with a mental one. However, a change in presentation style while preserving the isomorphic structure may result in a more subtle substitution of one mental process by another mental process. For example, in the Matrix game, a player may be mostly reliant on top-down processes (arithmetic operations), while in Marble Drop game, a player may also leverage from faster visual bottom-up processes (color perception).

All of the above examples show that problem solving is dependent both on the isomorphic structure of a task and its presentation style. Furthermore, a study of human behavior in a problem solving task should be done with respect to both the overall strategy dictated by its isomorphic structure and the individual cognitive processes imposed by its presentation style. However, the extent of the dependency of problem solving on presentation style is still to be investigated.

## Research Objective

The main question in this particular study is how the high-level strategy adapts to the perceptual characteristics of a task. The simplest adaptation would be to keep the top-down strategy the same. However, in such a case, some of the processes done originally by low-level perceptual processes should be transferred to top-down cognitive processes (or vice versa).

We are capitalizing on previous work [8] done on the game of SET (SET is a game by Set Enterprises, www.setgame.com) that provides a more or less complete description of the strategy players use. In SET, the rules and the isomorphic structure of the game largely determine the players' top-down strategy. However, the perceptual elements of the game can have a significant impact on how the strategy is implemented. This makes SET uniquely suited for our study of the effects of changes in presentation style at levels of both the overall strategy and the cognitive processes.

**Figure 2:**
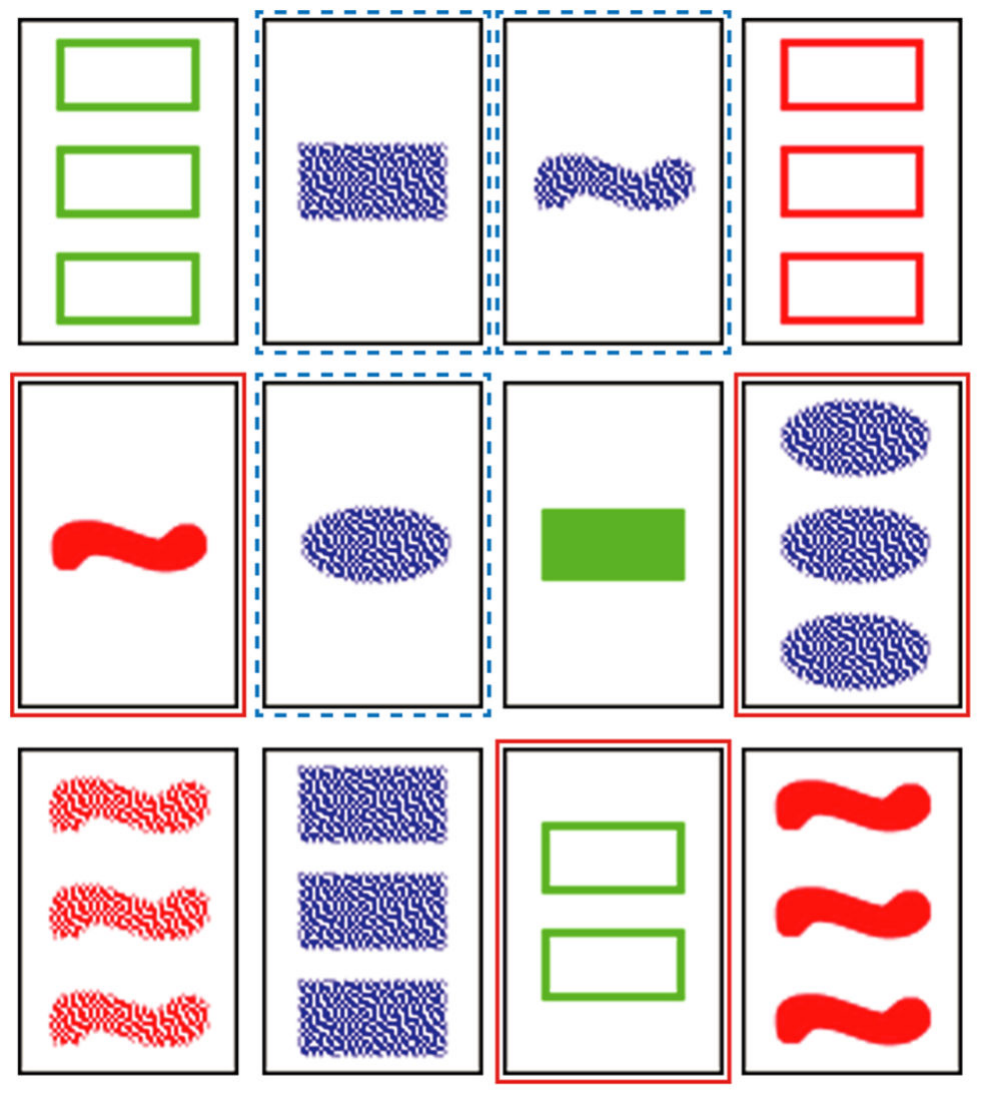
An example array of 12 set cards. The cards with the solid highlight form a level 4 set (all attributes are different), and cards with the dashed highlight form a level 1 set (Shape is different, and all other attributes are the same).

The SET card deck consists of 81 cards. Each card is uniquely defined by a combination of four attributes: color, shape, shading and number of shapes. Each attribute can have one of three distinct values: red, green, and blue for color; open, solid and textured for the shading; one, two and three for the number; oval, rectangle and squiggle for the shape. The rules for SET are relatively simple. At any moment in the game, 12 cards are dealt face up (Figure 2). From those 12 cards, players should find any combination of three cards, further referred to as a set, satisfying a rule stating that in the three cards the values for each particular attribute should be all the same or all different. We refer to the number of different features in a set as the *set level*. SET is quite a competitive game, since a player has to find a set before other players do, and this adds a certain degree of strategy to how the game is played.

The earliest study with SET [9] found that the time required to find a set increases as the set level increases. A more controlled study in which subjects were presented with 12 cards with only one set in it also showed the same pattern [8]. The reaction times already show a tendency toward a strategy that finds a set with similar cards faster than a set with dissimilar cards.

Further studies revealed that a player's strategy can be divided into two phases of search: dimension-reduction and dissimilarity-based search. Jacob and Hochstein [10] showed that players often reduce the search space by looking at groups of cards that share at least one attribute value. They referred to it as a dimension-reduction, since players reduce the number of attributes that they need to compare. The choice of a group of cards is highly dependent on group size: larger groups have a higher probability to be chosen. Surprisingly, subjects need as little as 600ms to extract such complex visual information as identifying the largest group of cards sharing a common attribute value among 12 SET cards [11]. This search is very much dependent on the visual similarity of the cards. Nyamsuren and Taatgen [8] further found that the choice of an attribute value for dimension reduction is not random, because players often prefer color over any other attribute. Furthermore, dimension reduction is mostly used at the beginning of the search, and, if a set cannot be found, players gradually transition to looking for increasingly dissimilar cards.

When players fail to find a set using dimension-reduction, they switch to dissimilarity-based search [8]. Dissimilarity-based search is used for finding higher-level sets with dissimilar cards. Players still focus on a particular attribute to guide the search. However, instead of looking at cards with the same attribute value, their attention is drawn to cards that have different values for the chosen attribute. Dissimilarity-based search does not allow the use of lower-level similarity-based perceptual processes. One can argue that discriminating between two colors can be done purely with bottom-up visual processes. However, identification of three colors that are all different from each other likely requires some form of top-down control. These factors make dissimilarity-based search a cognitively more demanding process. 

The strategy already provides clues about the type of cognitive processes involved. At the beginning of the game, subjects use perceptual processes to identify similar regions of the scene. Those processes are fast, efficient and more suitable for finding lower-level sets with similar cards. At the latter stages of the game, subjects use a slower, but more deliberate and controlled search to find higher-level sets. The preference for dimension reduction explains why subjects need less time to find lower-level sets than higher-level sets.

 The question remains whether the preference toward similarity is a result of a deliberate strategy choice or an effect imposed by presentation style. The iconic nature of the presentation style in SET makes it easy to identify similar cards using low-level perceptual processes. This advantage may prompt players to choose dimension-reduction over the more demanding dissimilarity-based search. It certainly can explain why players prefer to start the game with dimension-reduction and require less time to find lower-level sets. As such, presentation style may be directly influencing strategy choice. On the other hand, it is still possible that strategy choice is not dependent on presentation style and may be inherent to the structure of the task. The simplest way to test this hypothesis is to change the presentation style in such a way that the identification of similar and dissimilar groups of cards requires an equal effort. Of course, the task structure should be preserved. If strategy choice in SET is indeed defined by presentation style, then the preference for dimension-reduction should disappear. In other words, players should be equally likely to use dimension-reduction and dissimilarity-based searches at the beginning of the game. It is also possible that a new presentation style may even result in a new strategy. However, if strategy is defined by task structure, then we should observe little change in strategy, even if the presentation style of a task has been changed.

In this study, we used a modified version of SET, in which each card has a set of four words describing its four attribute values. The objectives and rules of the game are the same as in the original version. Word set is isomorphic to the original version of the game. However, the textual representation of cards removes most of the advantages inherent to perceptual components of the game. For example, textual representation should effectively deny subjects the ability to quickly identify a group of similar cards reported in [11]. On the one hand, it is interesting to analyze how problem-solving strategies change based on changes in presentation style. On the other hand, it might be the case that the strategy is still the same, and subjects prioritize similarity, despite the absence of a perceptual leverage. In this second case, the question is how cognitive processes are changed and adapted to apply the strategy to different visual presentations.

In our previous studies [8,12], we have described a cognitive model of a SET player. The model simulates a player's behavior at the level of individual cognitive processes involved during the game. Those processes include both high-level planning and visual bottom-up perception. The model uses the same strategies described earlier, and maintains an overall top-down cognitive control over the implementation of the strategy. However, individual steps within the strategy are highly dependent on low-level visual processes. For example, bottom-up activation from visual memory plays a key role in the model's choice of using either dimension-reduction or dissimilarity-based search. Using a new experiment, we can verify whether the model is still valid if most of the bottom-up aspects of perception are taken away. Additionally to providing a certain validation for the theories proposed in the paper, the model can also serve as a useful exploration tool. If players apply different strategies in word set, the model can help to investigate the cognitive processes underlying the new strategies including the primary triggers of strategy shift.

## Methods

### Ethics

The Ethical Committee Psychology (ECP) of the University of Groningen approved this study. Written informed consent as approved by the ECP was obtained from each participant before conducting the experiment.

### Subjects

In total, 20 subjects participated in the experiment. All subjects were students of the University of Groningen. The subjects' previous experience with SET ranged from a few played games to several years of experience. The results from two subjects were excluded from analysis due to extreme noise in the eye movement data.

### Design and procedure

**Figure 3:**
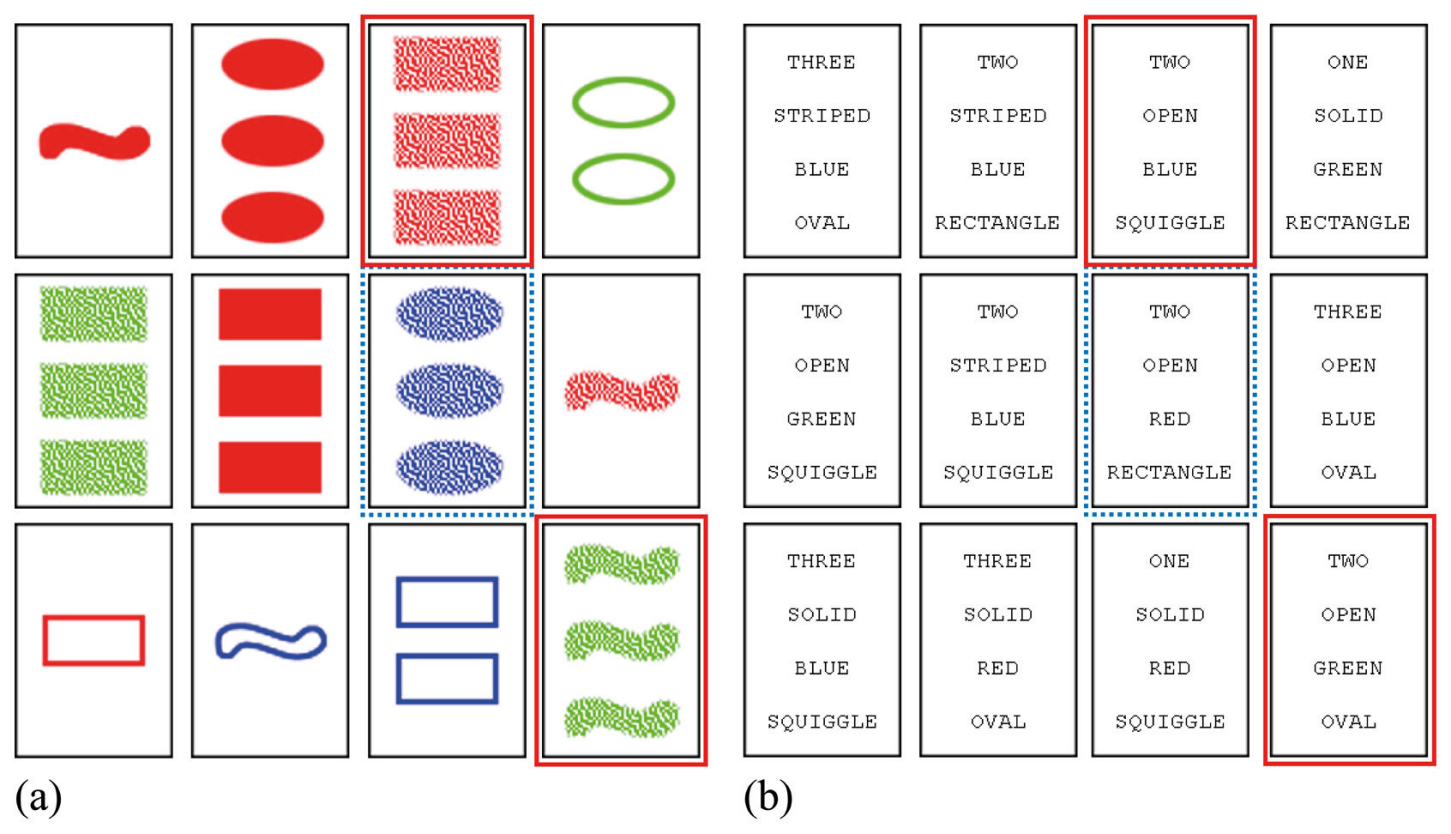
An example of a picture trial (a) used in the experiment and its equivalent word version (b). Cards highlighted with a border are the cards that form set (not visible for subjects). The card with a dashed border is a highlighted card.

The experiment was divided into two blocks with different trial types: a block with picture SET trials, and a block with word SET trials. Each block had 32 trials presented to subjects in random sequence. Each trial consisted of 12 cards shown on a computer screen and arranged in an array similar to the one in Figure 3. A trial had exactly one combination of three cards that formed a set. As a hint to the subjects, one of the set cards was highlighted by a red border. All trials were generated semi-randomly ensuring a same number of trials per difficulty level in each block. The order of the four attributes in each word SET trial was chosen semi-randomly from the following four possible combinations: (Shading, Shape, Number, Color); (Number, Shading, Color, Shape); (Color, Number, Shape, Shading); (Shape, Color, Shading, Number). It was ensured that all four of the combinations received an equal number of trials. Ten subjects started the experiment with a block of word trials, and eight subjects started the experiment with a block of picture trials.

The trials were essentially the same in the two blocks, except that attribute values were rotated between two blocks. Subjects were not told of this similarity. For example, while converting a picture trial into a word trial, all greens were replaced with blues, blues with reds and reds with greens. In a similar way, the values for other three attributes were rotated as well. This ensured that the trials in the two blocks were the same, but not recognizable by the subjects as such.

Prior to the experiment, subjects were asked to do six warm-up trials, three from each block, to let them become familiar with the experimental setup and with picture/word SET. The results from those trials were not included in the analysis. Half of the subjects started the experiment with a block of picture set trials, while the other half started the experiment with a block of word set trials. 

An EyeLink 1000 eye tracker was used for recording the eye movements. It is a desktop-mounted remote eye tracker with a monocular sampling rate of 500Hz and a spatial resolution of < 0.01° RMS. The card images were shown on a 20-inch LCD monitor with a screen size of 1024×768 pixels and a screen resolution of 64 pixels/inch. The card images had a size of 124×184 pixels, or 4.02°×5.95°. The horizontal and vertical distances between the images were 800 and 70 pixels respectively, which constitutes to 2.59° and 2.27°. Angular sizes were calculated with an approximate viewing distance of 70 centimeters since the subjects were given a certain freedom for head movement. The gaze position, as calculated using the eye's corneal reflection captured with an infrared camera, compensated for head movements. The eye tracker's default parameters were used to convert gaze positions into fixations and saccades. The calibration of the eye tracker was performed at the start and during the experiment, if necessary. A calibration accuracy of 0.8° was considered acceptable. Before each trial, subjects were asked to do a drift correction as an additional corrective measure.

## Results

This section provides an analysis based on the subjects' reaction times and eye movements. Most of the eye movement analyses are based on collapsed fixation sequences in which consecutive fixations of the same card are collapsed into one fixation. It is explicitly mentioned when raw fixation sequences have been used in the analysis.

### Reaction times

The reaction times provide the first clue about possible strategies used in the two types of the game. According to a mixed effects two-way ANOVA done on log-transformed reaction times, the order of two blocks had no significant effect on the subjects' overall reaction times (*F(1, 16*)*=1, p=0.331*), nor did it have a significant effect on reaction times in either picture or word trials (*F*<1). Reaction times in word trials were significantly higher than reaction times in picture trials (*F(1, 16*)*= 158.913, p<0.0001*), independently of the order of blocks.

**Figure 4:**
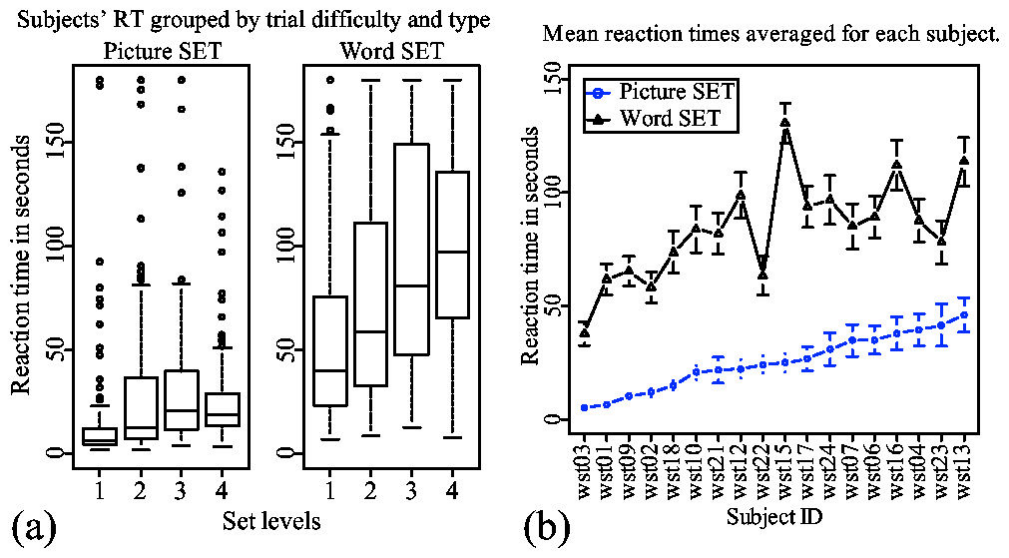
Mean reaction times averaged for different groups. (a) Reaction times grouped by trial difficulty and type. (b) Each subject's mean reaction times averaged in two trial types.

In Figure 4, median reaction times for picture trials show the characteristic increase of RT as a result of an increased SET level. We used a mixed-effect linear regression analysis on log-transformed reaction times with set level as a predictor and subjects as a random effect. The analysis showed that reaction time increased on average by 4.54 seconds as the set level increased (β = 0.2939, *t = 9.774, p < 0.001*). This effect is similar to the results from previous studies [12].

Reaction times for word trials also exhibit the same effect. However, subjects needed more than twice the amount of time to find sets in word trials than in picture trials. An identical mixed-effect linear regression analysis indicated that reaction time increased on average by 17.2 seconds as the set level increased (β = 0.2591, *t = 10.44, p < 0.001*). There was a positive correlation between the subjects' mean reaction times in picture and word trials: *r(16*)* = 0.66, p < 0.01*. This indicates that subjects who perform well on finding picture sets can be expected to be good at finding word sets as well.

Overall, subjects are better at finding sets with similar cards in both types of the game. It is therefore likely that subjects are using dimension-reduction not only in finding picture sets, but also in finding word sets.

### Card encoding

The difference in encoding processes can have a significant effect on how information is stored in working/long-term memory, and on how it is further processed. For example, if a card was encoded as a series of visual objects rather than a single object, then it is likely that it will be processed and stored in memory as a series of visual objects.

**Figure 5:**
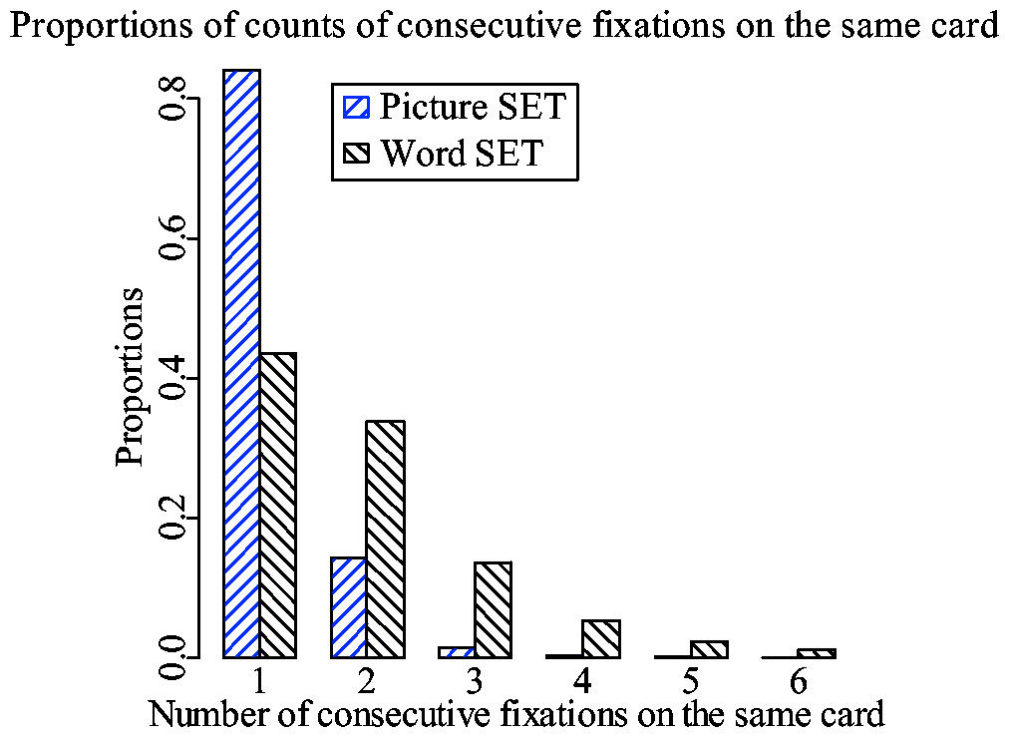
Proportions of the counts of consecutive fixations on the same card. Proportions have been calculated separately for picture and word trials.

There is a difference between picture and word trials in terms of the number of fixations required to encode a card. It is hard to quantify exactly how much information about a card is encoded at each instance. However, it is safe to assume that during a fixation in a picture trial, a subject encodes at least as much information as during a fixation in a word trial. Figure 5 shows how many consecutive fixations subjects need to encode a card. The proportions were calculated from raw fixation sequences. In picture trials, subjects need one fixation 84% of the time. However, in word trials, occurrences of one fixation per card amount to 43%. Often subjects need two or more fixations to encode a card. This suggests that there is quite a significant difference between picture and word trials in terms of the effort required to encode a card. In word trials, subjects ideally need four fixations, one fixation per attribute, to encode an entire card. Furthermore, in around 4% of the time, subjects had more than four consecutive fixations on the same card. The results suggest that, in word set, a card is encoded as a series of visual objects, as opposed to the single coherent object encoded in picture set.

With respect to picture set, we should be careful to claim that the process of encoding a card is not holistic, even if it results in a coherent visual chunk. Holistic recognition is unlikely because card encoding in picture SET does not violate any of three principles defined by the General Recognition Theory [13,14]. It is more likely that card encoding is a hierarchical process in which individual attributes are encoded first and then combined into a coherent object. 

Processing of word set cards is definitely not holistic. Holistic perception requires a visual object to have a sufficient acuity relative to its distance to the focal point. Text has one of the lowest acuities among common feature dimensions. Kieras [15] defined a visual angle of one degree as the distance to the focal point within which individual letters are recognizable. Therefore, even if all three GRT principles were violated, holistic recognition of word set cards would be impossible due to the physical limitation of acuity.

The remaining eye movement analyses in this article were based on collapsed fixation sequences, in which consecutive fixations of the same card have been collapsed into one fixation.

### Dimension-reduction and dissimilarity-based search

**Figure 6:**
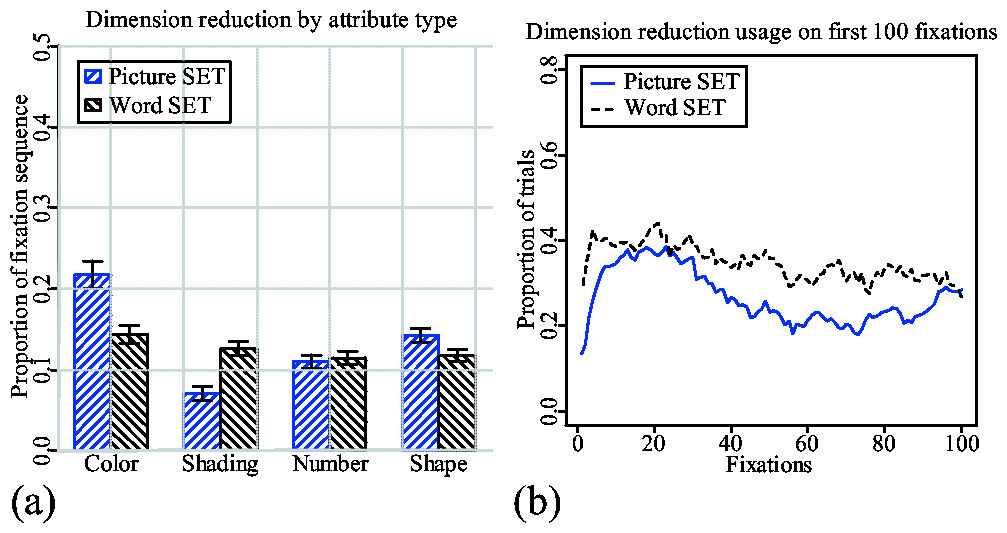
Subjects' overall usage of dimension reduction in two trial types. (a) The usage of attribute types in similarity-based scanning as a proportion of the trial's fixations sequence. (b) The probability of using dimension-reduction based on the fixation's position within a trial. The probability at fixation *x* is calculated as: *N*(dr(*x*))/*N*(*x*). *N*(dr(*x*)) is a number of trials that have dimension-reduction blocks that include fixation *x*; and *N*(*x*) is a total number of trials that have at least x number of fixations. Fixation sequences in word trials are significantly longer than in picture trials. For comparison purposes, fixation sequences and dimension-reduction blocks from word trials were transformed into shorter lengths to match the lengths of corresponding picture trials.

We have calculated the usage of dimension-reduction from eye movement data using the same methods described in our earlier study. This method finds blocks of consecutive fixations on cards that have at least one common attribute value. Next, all blocks that have a chance probability above 0.05 were filtered out. Since each card has four attributes, there can be overlapping blocks within the same subsequence of fixations. Overlapping has been removed by cutting the right-most blocks at the point of overlap. The chance probability has been recalculated for the leftover blocks. Finally, the lengths of the resulting significant blocks were used to calculate the proportions shown in Figure 6a. Due to its complexity, we would like to refer to the original study for more details on the calculation method [8].

Figure 6a shows how often each attribute is used in dimension-reduction during the course of a single trial. Fixation sequences for the trials in which dimension-reduction is impossible with respect to a particular attribute value were removed from analysis. For example, if the highlighted card is green and there are only two other green cards, then the trial is not used for calculating a proportion of dimension-reduction by color. The proportions were calculated separately for picture and word trials. For example, the first bar in Figure 6a shows that 22% of the fixation sequence of a picture trial will be occupied by fixations in which the subject did dimension-reduction by color. As with the previous study [8], in picture trials, subjects show a clear preference to color over any other attribute. However, in word trials, there is hardly any preference to any of the attributes with nearly equal proportions on each attribute type.

Figure 6b shows how likely it is that dimension-reduction will be used during the first 100 fixations of the trial. Again, the proportions are shown separately for picture and word trials. The probability that subjects will use dimension-reduction during first the 30 fixations of a picture trial is, on average, around 40%. The probability then goes down with each consecutive fixation. The mixed-effect linear regression analysis done on proportion lines calculated for individual subjects shows that this decrease is significant (Table 1). This analysis used all fixations in positions between 20 and 80. This decreasing pattern is, again, very similar to one found in the earlier study [8].

**Table 1:** The result of a linear mixed-effect regression analysis of a predicted proportion of dimension reduction based on a fixation position and a trial type.

	Estimate	Std. Error	*t* value	*p* value
Intercept (Picture trial)	0.3866	0.0169	22.88	< 0.001
Fixation position	-0.0028	0.0002	-13.84	< 0.001
Word trial	0.0376	0.0152	2.48	0.013
Fixation position and word trial interaction	0.0011	0.0003	3.86	< 0.001

Dimension-reduction also occurs frequently in word trials. In addition, the main and interaction effects of the fixation positions shown in Table 1 indicate that there is an overall slow, but significant decrease in the proportion of fixations devoted to dimension reduction as a trial progresses. This indicates that subjects are also using dimension reduction in word trials, although with no preference toward a particular attribute value. The visible difference between the two probability lines in Figure 6b can be explained by different scanpath structures imposed by differences in visual presentation. This issue is explored further using model simulations. The reader can also refer to Materials S1 for additional analysis based on ARIMA models applied to data on Figure 6b.

Dimension reduction is a similarity-based strategy. A player searches for a set among cards that are similar with respect to, at least, one attribute dimension. However, subjects gradually stop using dimension-reduction and start looking for higher-level sets. This means that subjects start searching for a set among cards that are increasingly dissimilar. This pattern can be revealed by dividing a trial's fixation sequence into consecutive series of subsequences, and by calculating the overall similarity of each subsequence to the highlighted card.

An earlier study with SET [8] has shown that subjects refixate on a highlighted card approximately every five fixations, presumably to refresh their memory and to restart a new search subsequence. For example, the following labeled fixation sequence “4-7-11-10-3-7-2-11-4-3-10-2-5-9-5-6-4-7-5-8-4”, with 4 being a fixation on a highlighted card, can be broken down into three subsequences. Next, each subsequence's overall similarity to the highlighted card can be calculated.

**Figure 7:**
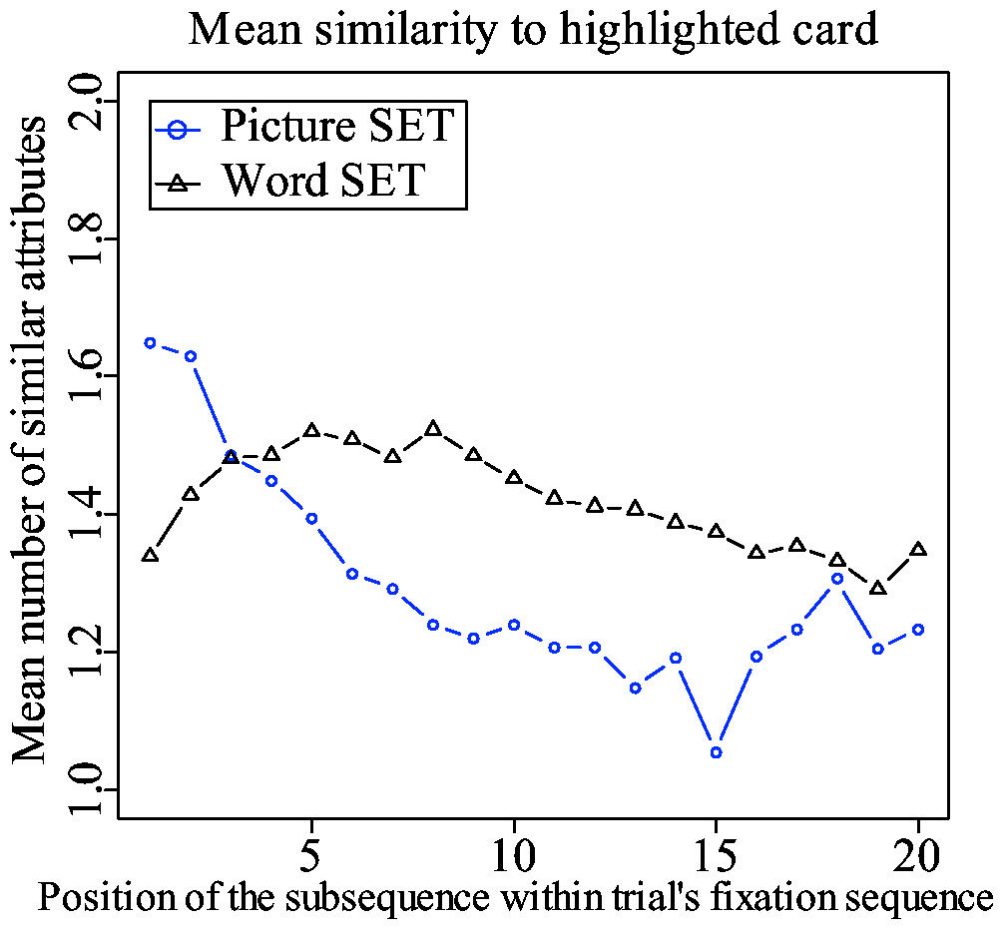
The mean overall similarity of all cards in a particular subsequence to the highlighted card. The values are calculated separately for picture and word trials.

The same subsequence-based analysis was done in this study. As shown in Figure 7, the mean similarity of fixated cards to the highlighted card decreases over time in picture trials. A linear mixed-effect regression analysis done on the first 20 subsequences indicates that the decrease is significant (the main effect of subsequence's position on Table 2). The decrease is very similar to the one found in previous study [8]. The same effect is also present in word trials. However, the decrease in similarity, although significant, is very slow (the interaction effect on Table 2). This slight decline is nowhere near as big as in picture trials.

**Table 2:** The result of a linear mixed-effect regression analysis of a predicted similarity to a highlighted card based on a subsequence's position and a trial type.

	Estimate	Std. Error	*t* value	*p* value
Intercept (Picture trial)	1.474	0.015	100.3	< 0.001
Subsequence position	-0.020	0.001	-18.9	< 0.001
Word trial	-0.009	0.014	-0.65	0.518
Subsequence position and word trial interaction	0.013	0.001	9.13	< 0.001

In picture set, subjects are clearly transitioning into a dissimilarity-based search as their trials progress. However, the same effect is not conclusive in word set. Nevertheless, considering that subjects were able to find level 4 sets, it is reasonable to assume that dissimilarity-based search was applied in word set trials, despite the lack of evidence in eye movement data.

### Systematic versus unsystematic scanpaths

Both dimension-reduction and dissimilarity-based strategies require visual searches. The spatial characteristics of the scanpaths can give insights into differences in visual searches between the two types of tasks. 

**Figure 8:**
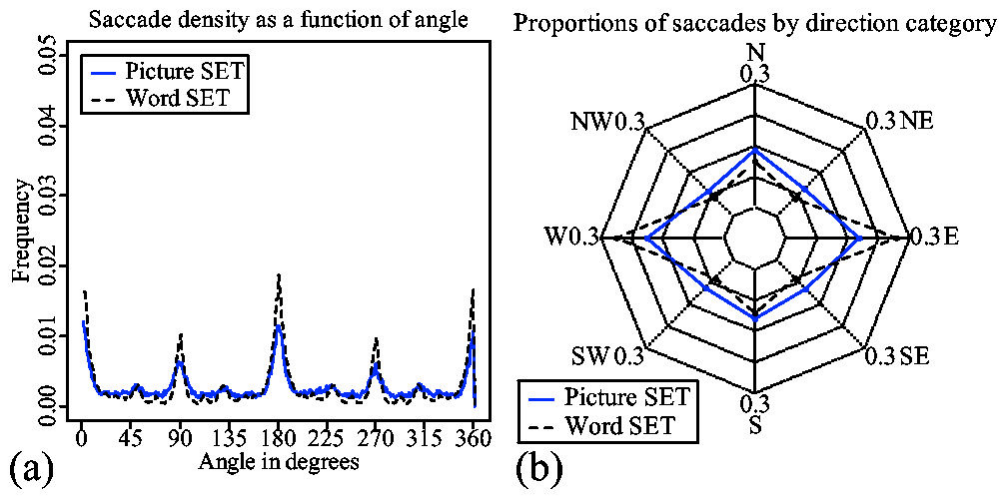
A radar chart for the proportions of saccades in each saccade category.

Figure 8a shows a density plot based on the saccades' raw angles. The plot reveals four very distinct distributions centered around 0/360, 90, 270 and 360 degrees. It indicates that in both picture and word trials, subjects prefer to make horizontal and vertical saccades. Such a preference can be partially explained by the grid-like presentation structure of the scene. However, higher peaks in distributions of word trials indicate that preference for vertical and horizontal saccades might be higher in word trials. This difference cannot be accounted for by presentation structure, since this structure is identical in both types of trial.

Ponsoda, Scott and Findlay [16] proposed to measure the systematicity of visual search based on the proportion of diagonal saccades. The higher the proportion of diagonal saccades is, the less systematic the search. Figure 8b shows a radar chart with the proportions of saccades in each of the eight direction categories defined in [16].

Firstly, the logistic mixed-effect regression analysis indicates that there is a significant difference in proportions of diagonal saccades made in word and picture trials. The probability of a diagonal saccade in a picture trial is 0.34 (the intercept on Table 3). The same probability in a word trial decreases to 0.25 (the negative main effect of word trial on Table 3). The low probability of diagonal saccades indicates that visual search is, in general, systematic in both types of trial. The decrease in diagonal saccades in word trials indicates that subjects are less systematic in picture trials than in word trials. This difference in systematicity may account for the differences in distribution shown in Figure 8a.

**Table 3:** The results of a logistic mixed-effect regression in which the predicted value is the probability of a diagonal saccade.

	Estimate	Std. Error	*z* value	*p* value
Intercept (Picture trial)	-0.676	0.036	-18.79	< 0.001
Word trial	-0.420	0.035	-12.03	< 0.001
Trial level	0.046	0.010	4.69	< 0.001
Word trial and trial level interaction	-0.035	0.012	-2.96	0.003

Next, there is a small, but significant effect of trial level on the probability of a diagonal saccade. Subjects are more likely to make diagonal saccades in more difficult picture trials (the positive main effect of Trial level on Table 3). However, this effect is greatly reduced in the word trials (the significant negative interaction effect on Table 3).

The results suggest that in word set, subjects do more structured scannings with more prevalent horizontal and vertical saccades than in picture trials. The increased systematicity of the scanpaths in word set may be related to the lack of visual clues in peripheral regions to guide visual attention. As a result, subjects may be forced to do exhaustive searches in word set, as opposed to more guided searches in picture set. Such exhaustive searches can also explain the lack of evidence for dimension reduction and dissimilarity-based search in the analysis of eye movements from word set trials.

## Discussion

### Strategy in picture trials

Evidence from the experiment indicates that there is a gradual shift from dimension reduction to dissimilarity-based search in both versions of the game. As discussed in the previous study [8], individual steps within a strategy are the same for both dimension-reduction and dissimilarity-based search. Searching for a set is a repeated comparison of three cards. Therefore, a player's strategy ultimately boils down to finding an optimal way to decide which three cards to compare. The subject is already provided with a highlighted card, so he picks a second card and then searches for a third card that may form a set with two already selected cards. If he cannot find a suitable third card, then he picks another card as a second card and starts a new search for a third card. The choice of a second card depends on preferences toward attribute types, and on whether dimension-reduction or dissimilarity-based search is being used. For example, at the beginning of a trial, a subject is more likely to choose a second card that is similar to a highlighted card, since dimension-reduction is preferred at this point. Furthermore, it is more likely that the second card shares the same color with a highlighted card than, for example, the same shading. However, over time, the choice of a second card is geared toward less similarity to a highlighted card. The entire strategy is simple, but effective enough, and, simulated in a cognitive model [8,12], gives the same pattern of behavior as exhibited by human subjects.

### Strategy in word trials

Based on an initial impression, it appears that subjects are using different strategies in picture and word trials. However, we propose that the strategies are the same. This assumption is supported by a significant positive correlation between the subjects' reaction times in word and picture trials. Furthermore, the fact that subjects need more time to find higher level sets than lower level sets in word trials as well suggests the same bias toward similarity as was found in picture trials. It also supports the assumption that the strategies are the same. Increased reaction times in word trials and other changes in behavioral data can be accounted for by a poor quality of visual information that leads to different cognitive processes being used for strategy implementation. The lack of a visual acuity of an attribute value presented as a text has several implications in terms of different cognitive processes involved in the two types of trials. 

#### Scene gist

The lack of visual acuity in word trials hugely affects subjects' ability to leverage from peripheral vision. In picture trials, attribute values are mostly identifiable in peripheral vision, and a subject can catch the gist [17,18] of a scene almost instantaneously. Such a gist is used for guiding attention and for encoding specific objects in the scene. If one is looking for a green card, then it is almost immediately obvious where all of the green cards are. In word trials, text is not identifiable in peripheral vision. So the gist that is readily available in a picture trial is absent in word trial. One could argue that in word trials subjects can gradually build up the gist of the scene in visual short-term memory after several initial fixations. However, such a gist will be extremely complex and unpractical, since every card is encoded as a collection of four objects. In addition, visual memory has relatively short temporal persistence, usually within a few seconds [19].

#### Card encoding

There is a difference between picture and word trials in terms of how information about the card is stored in memory once it is encoded. Previously, it was mentioned that in a word trial, a subject needs more than one fixation to encode a card (Figure 5). In picture set, a subject fixates on a card and encodes it into a single coherent visual object. This object contains information about all four of the attributes of the card. However, in a word trial, a subject ideally needs four fixations, one fixation per attribute, to encode an entire card. Moreover, the card is encoded not as a single coherent object, but as a collection of four different visual objects, and is subsequently stored in a memory as such. This introduces an additional overhead of associating four objects with a single card.

#### Dimension-reduction

The fact that reaction times for lower level sets are shorter than reaction times for higher-level sets (Figure 4a), indicates that dimension-reduction is still being used in word trials. The slow, but steady decrease in Figure 7 also indicates to a certain preference for similarity-based search. However, the absence of a gist has a significant influence on how a subject does dimension-reduction. There are several studies indicating that different features are not equally identifiable in peripheral vision [19,20]. For example, it is easier to identify color than any other feature. Hence, color is present more prominently in a gist, and subjects are more likely to choose color for dimension-reduction [8]. However, absence of a gist in a word trial removes preference for any particular attribute. This is the primary reason why Figure 6 shows very little difference between attributes in word trials.

#### Scanpaths

**Figure 9:**
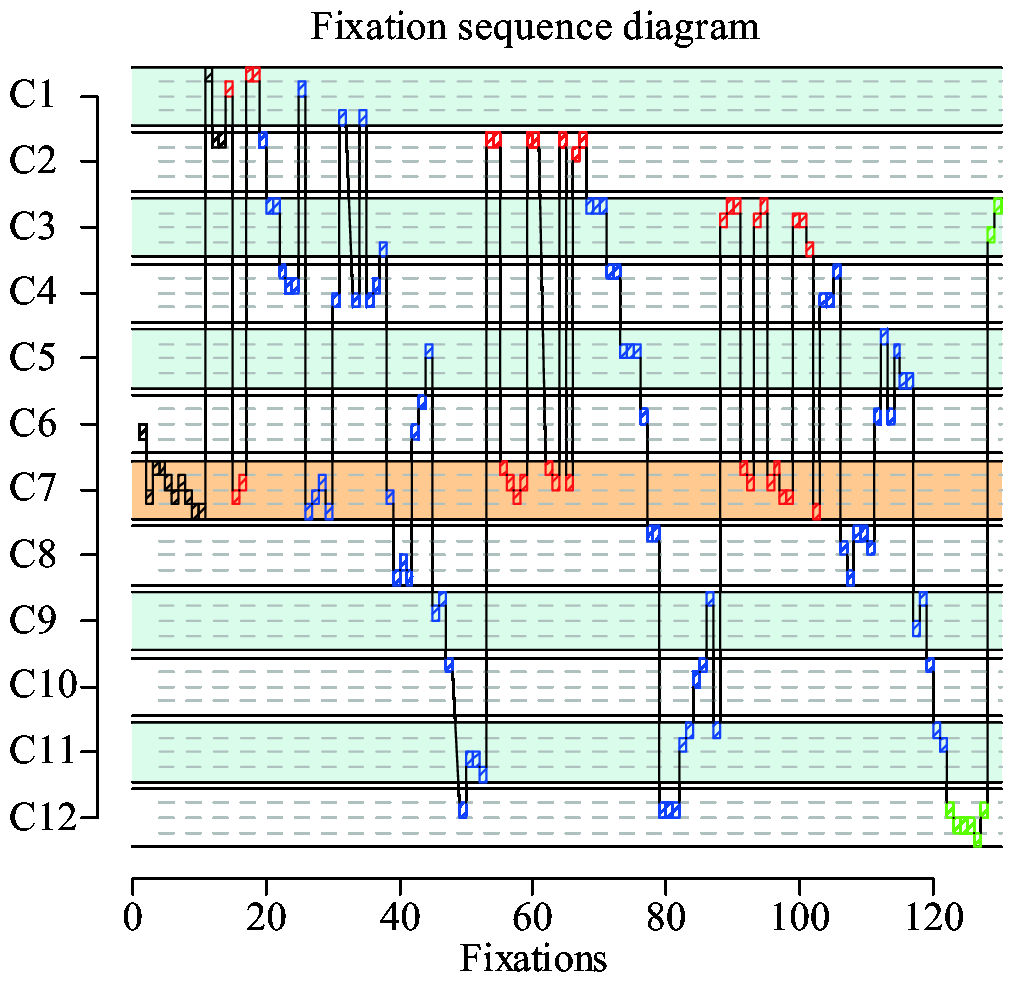
An example of an annotated raw fixation sequence produced by wst03 during the trial shown in Figure 3a. Each lane with solid boundaries represents a card, whereas each sublane with dashed boundaries represents an attribute within a card. The lane labeled as C7 is the highlighted card. The other two cards that belong to the set are C3 and C12. Each rectangular block represents a fixation on a card's attribute value. Red blocks represent fixations where a subject paired a highlighted card with another card, while blue blocks represent consecutive search for a third card. Green blocks are fixations where the subject found a set and made final verifications.

As we have discussed earlier, in a picture trial, a subject tends to pair a highlighted card with a second card and then searches for a third card that can potentially form a set with the pair. The same strategy is applied in word set. Figure 9 shows a very nice example. It is a scanpath produced by one of the subjects during the trial shown in Figure 3a. As the scanpath indicates, the subject probably formed at least two pairs during the course of the trial. Repetitive back and forth fixations (between 50 and 70 fixations) between the highlighted card C7 and the second card C2 indicate that a pair was formed out of these two cards. Next, the subject scans for a matching third card up until 90th fixation. Since the subject was not able to find a matching card, the new pair was formed with card C3 (between 90 and 103 fixations). A new search for a matching card was done up until 122th fixation, where the subject identified card C12 as a possible match. Indeed, cards C7, C3 and C12 form a valid set, so trial finishes.

Although these are the same basic steps as in a picture trial, there is one significant difference between scanpaths. The search for a third card in picture set is supported by a scene gist. If a subject is looking for a green card, then it is immediately identifiable where all of the green cards are. However, in word set there is no gist to make such targeted attention shifts. Instead, a subject needs to fixate on every single card to check whether a card has desired attribute values. Indeed, the searches for third cards shown in Figure 9 are very much exhaustive.

This difference between exhaustive and targeted searches explains why scanpaths in word trials have less diagonal saccades than scanpaths in picture trials. So what appears to be a systematic visual search might rather be a search done out of necessity due to lack of proper visual features to aid the search in peripheral regions. Similarly, abundance of diagonal saccades in a picture trial is not the result of an absence of systematicity as would be suggested in [16]. It is rather the result of subjects taking a targeted "shortcut" by using visual features that can be processed by peripheral vision during a systematic search.

### Types of visual search

There are several competing explanations of how humans accomplish visual search tasks. Early studies of visual search suggested that visual search may be sequential (only one object is encoded at a time), because of the positive slope produced by the RT×set size function. The visual search observed in the set tasks is clearly not sequential. As was discussed earlier, subjects need as little as 600ms to extract such complex visual information as identifying the largest group of cards sharing a common attribute value among 12 SET cards [11]. This result clearly refutes the possibility that visual search is a purely sequential search. There are certainly some parallel processes involved.

An alternative explanation of sequential search is a limited-capacity parallel search [21]. In this paradigm, several visual objects can be encoded at the same time, but the number of objects is limited by the capacity of the visual process. It is highly unlikely that limited-capacity visual search is used in set tasks. SET belongs to a group of tasks under the comparative visual search (CVS) paradigm. Previous studies showed that the eye movement patterns in CVS tasks show clear signs of well-structured sequential search [22]. Eye movement data from the two set tasks also suggest that searches are not parallel. For example, just like other fixation sequences, the fixation sequence shown in Figure 9 exhibits signs of a highly structured sequential search. In another example, we found subjects often end the trial with verification fixations. Verification is characterized by repeated back and forth fixations on three cards forming a set. Such fixations were observed in both picture and word set trials. Such fixations would not have been necessary in limited-capacity parallel searches. Similar verification fixations were also observed by Pomplun et al. [22].

As a third alternative, Wolfe proposed that all visual search tasks require the deployment of attention to the target, but such a deployment is guided by pre-attentive parallel processes [23–25]. Furthermore, Wolfe suggested that search tasks only vary with respect to the degree in which they can use parallel processes to deploy attention. It is quite likely that the visual search used in the set tasks follows Wolfe's theory. It certainly explains why subjects can quickly capture scene gists [1], but also exhibit sequential visual search behavior such as in Figure 9. Conformance to Wolfe's theory also adds additional credibility to our explanation of why visual search strategies are essentially the same in picture and word sets. Visual search is the same in the two tasks, but the use of parallel processes in word set is impaired by the poor acuity of the text.

## Cognitive Models

The major question we want to answer using cognitive models is whether the differences in cognitive processes that were described in the previous section can really account for the behavioral differences subjects have shown in picture and word trials. Our previous studies [8,12] have already described the cognitive model for picture set. For this study, we have reused the same model to simulate human behavior in picture trials. We have also developed a second model that does the word trials.

The two models are nearly identical. Both models use the same set of values for adjustable parameters and follow the same strategy of playing the game. The only difference lies in the processing requirements for the two types of cards.

### Cognitive architecture

**Figure 10:**
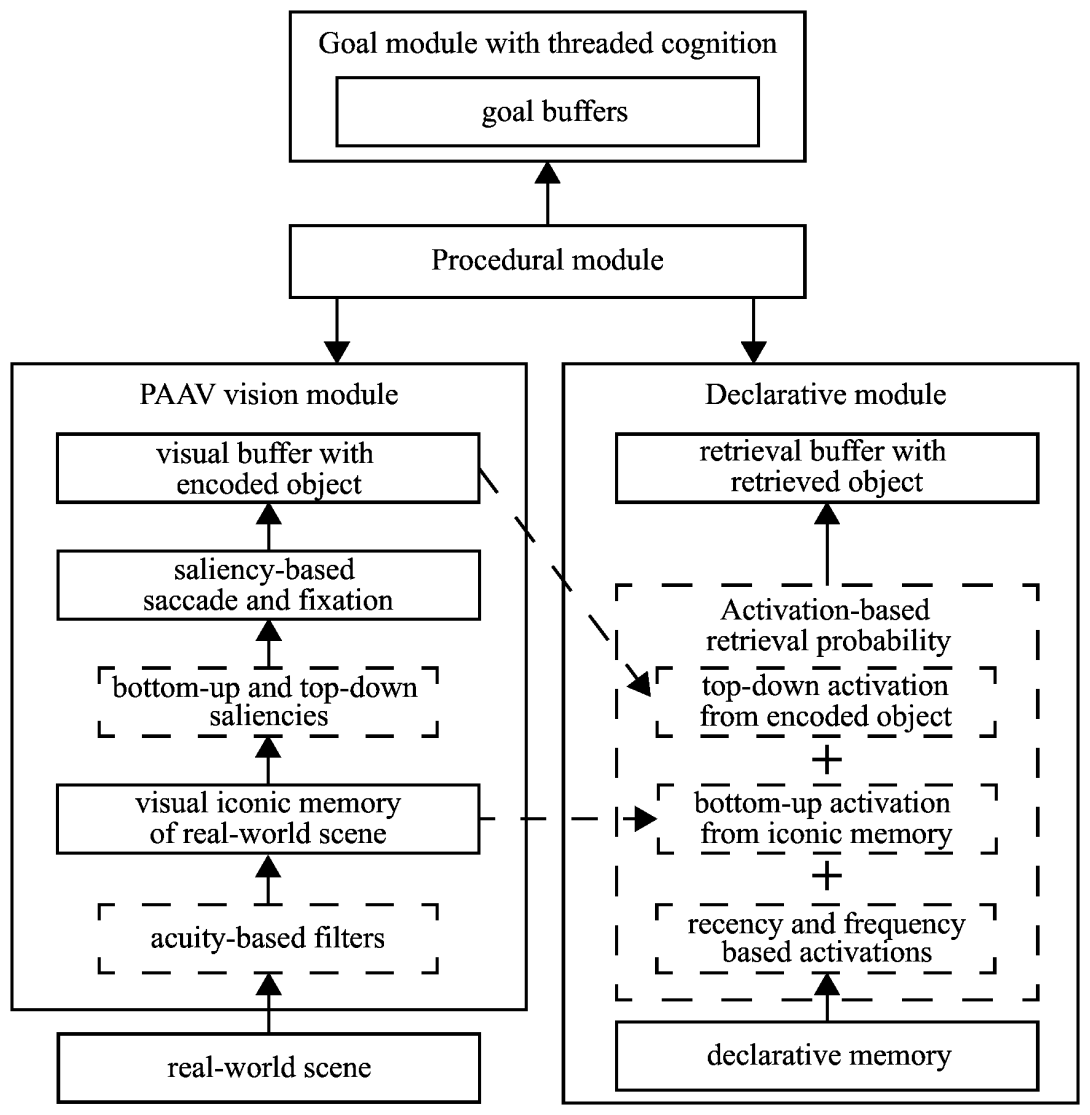
A simplified depiction of ACT-R architecture. Internal workings and external connections between vision, declarative, goal and procedural modules. These four modules provide the most of the functionalities necessary for modeling SET tasks.

We have used the ACT-R cognitive architecture [26] to develop the models. ACT-R consists of several modules, such as a Vision module for handling visual processing, a Declarative module for simulating declarative memory, and a Goal module for tracking the model's state and objectives. The modules mostly communicate with each other via the Procedural module, which allows the modeler to write task specific production rules. However, in limited cases, modules can also spread activation to other modules simulating low-level cognitive processes. Figure 10 shows the internal working of the most important modules in detail. A description of the figure will be provided next.

We used several extra modules that are not part of ACT-R by default. The extra module most important to the task is the Pre-attentive and Attentive Vision module [12] or PAAV for short. The PAAV module provides several functionalities that are otherwise not supported by ACT-R's default vision module. The other two extra modules are Threaded Cognition [27] and Base-Level Inhibition [28]. With Threaded Cognition, we assume that there are two separate and parallel meta-controls governing the overall top-down strategy and the bottom-up visual attention shifts, respectively. Lastly, the Base-Level Inhibition module provides a short-term activation inhibition of items in declarative memory. This module is necessary for modeling complex short-term tasks in which several alternatives need to be stored in and retrieved from memory.

#### Differential acuity

PAAV recognizes that not everything in a visual scene can be seen [by the model] at any given moment. Human vision is limited, especially in the extra-foveal region [29]. The further away an object is from a current focal point, the harder it is for the human visual system to recognize its features. Furthermore, different features, such as color or shape, have different acuities [19]. For example, color has a higher acuity than shape. This means that the visual system will be able to recognize the color, but not the shape, of an object that is in a certain distance from the foveal point. The PAAV module uses different acuity functions for color, shape, size and shading with color having the highest acuity. Text is also supported by PAAV in a sense that any word is treated as a shaded rectangular object of a same size as the word. However, there is a separate acuity function for recognizing the pattern of individual letters in the word. In order for individual letters to be recognized, a word should be inside the foveal region. PAAV considers this region as a circle with a radius of one degree of angular distance from the point of fixation. This estimation is provided in [19].

#### Scene gist and visual iconic memory

It is often reported that human vision can pre-attentively capture the gist of a visual scene [17,18]. This is a quick and parallel process that captures just enough details to further guide visual attention to informative parts of the visual scene for a finer grained analysis. The PAAV module also captures the gist and stores it in iconic memory. Iconic memory may contain complete information for some objects, such as an object's color, shape, shading and size. However, for most visual objects, iconic memory will contain incomplete information (e.g. color only) due to limited acuity. Furthermore, an object's features in iconic memory, despite trans-saccadic persistence, decay after a short period of time (currently 4 sec) if they are not accessible via peripheral vision anymore.

#### Attentional guidance

It is well known that the human visual system prioritizes parts of the visual scene for attentional capture [30]. This process is a combination of bottom-up and top-down guidance [31,32]. Bottom-up guidance draws attention to the parts of the visual scene that are most salient due to the inherent properties of the scene. For example, a single green card among red cards will draw attention due to a pop-out effect. On the other hand, top-down guidance draws attention to the parts of a scene that are relevant to the current task at hand. For example, if a player is looking for a green set, then all green cards will be prioritized for attentional capture, while all non-green cards will be inhibited. The PAAV module mimics this process by calculating top-down and bottom-up saliency values for every object in iconic memory and choosing the one with the highest overall saliency as the next point of attention.

#### Spreading activation from iconic memory

In ACT-R, knowledge chunks are stored in declarative memory. Each chunk has an activation value that reflects its recency and frequency of retrieval. The chunk with the highest activation has the highest probability of retrieval. However, it has also been observed that visual stimuli can influence the result of memory retrieval [33]. The PAAV module simulates this effect whereby each visual object in visual iconic memory spreads activation to every matching chunk in declarative memory. So, depending on the content of iconic memory, results from two identical retrieval requests can differ. ACT-R's default vision module also allows spreading activation from an encoded visual object to declarative memory, thereby simulating a more top-down influence.

### Model details

Any ACT-R model is essentially a set of production rules expressing task specific instructions. A production rule consists of a left-hand side condition part and a right-hand side action part. A production rule fires when all of the conditions in the right hand side are met. Only one production rule can fire at a time. For example, if the condition part says that the current goal of the model is to attend the highlighted card, then the action part tells the PAAV module to shift attention. The right-hand side action part can also set the goal of the model to a new one. The production rules in models of set tasks implement the strategy described next.

#### Strategy

Although we used two separate models, both of them use exactly the same strategy. This paper describes the strategy only on a level of details necessary to understand the inner workings of the model. Please, refer to [8,12] for a more complete description. The following is a description of the model’s general strategy:

1. Focus attention on the highlighted card *HC*. Let *Card*_*HC*_ be a set of four attribute values in the highlighted card.
2. Retrieve any attribute value *V*_*DM*_ from declarative memory with *A*_*V*_ being the attribute type of *V*_*DM*_.
3. Pick the attribute value *V*_*HC*_ from *Card*_*HC*_ that also has *A*_*V*_ as attribute type.
4. If *V*_*DM*_ = *V*_*HC*_, then use dimension-reduction by defining search space *G* as a group of cards that has *V*_*HC*_. If *V*_*DM*_ ≠ *V*_*HC*_ then use dissimilarity strategy by defining search space *G* as a group of cards that does not have *V*_*HC*_.
5. For every card *C2* in search space *G*, search for a third card *C3* that forms a set with *HC* and *C2*. If a set is found, then finish the trial.
6. If there is no more card *C2* to choose from search space *G*, then go back to step 1.

The critical step is step 2, in which a top-down influence (the highlighted card) and a bottom-up influence (the prominence of attribute values in iconic memory) determine what attribute value the model is going to pursue.

Although the two models use the same strategy, there are several essential points of difference that rise due to presentation differences. 

#### Visually encoding a card

The model for picture set can encode all four values of a card in a single fixation, since those values are perceived as four integral features of a single object. However, the model for word set has to fixate on each individual value of a card, since each word is treated as a visual object of its own. Therefore, instead of just one fixation, four fixations are needed just to encode all four values in a word set model.

#### Scene gist in visual iconic memory

**Figure 11:**
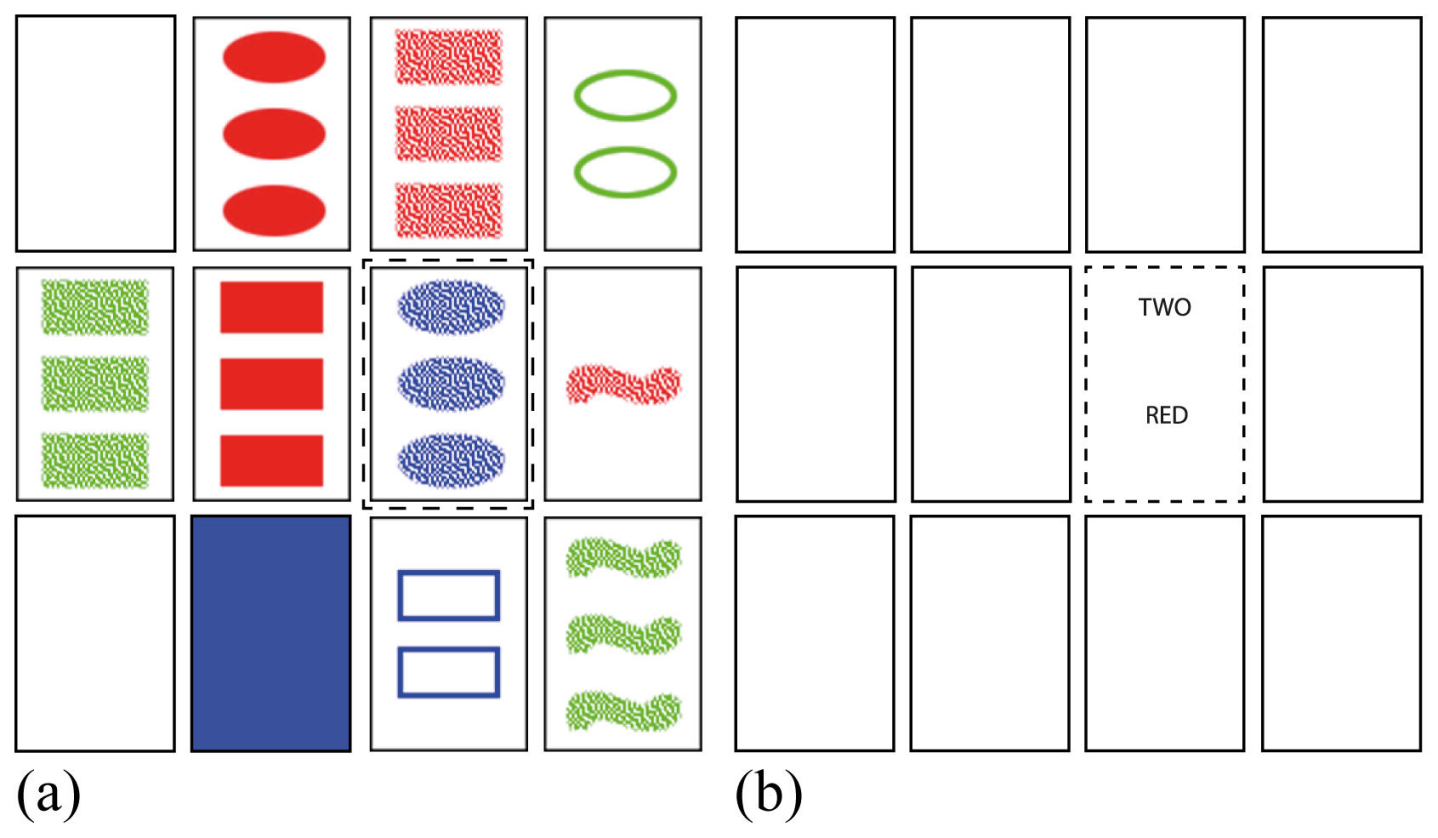
A visualization of the content of the model's iconic memory. Contents were visualized after the first fixations on the highlighted cards (cards with dashed boundaries) in (a) picture and (b) word trials. Those are the same trials as shown in Figure 3.

The model for finding picture sets has a reasonably detailed representation of the trial in its iconic memory from the start. The acuity limitation of a text prevents the model for word trials from building up iconic memory with the same level of detail. Figure 11 contrasts the contents of iconic memories of the two models after the first fixation on the highlighted card was made. Except for three cards on the left, the model for a picture trial has near complete information about the visual scene (Figure 11a) in its iconic memory. This information is enough to calculate both bottom-up and top-down saliencies for cards to guide attention shifts. The model for word trials has barely any information about the visual scene (Figure 11b). All it has is an encoded value "TWO" for the Number attribute and a pre-attentively recognized pattern of individual letters for "RED". There is no information to guide attention shifts from either bottom-up or top-down perspectives. 

#### Dimension-reduction

Both models have a tendency to use dimension-reduction in the early stages of a trial. Spreading activation from the encoded highlighted card biases the retrieval process. As a result, values that belong to a highlighted card have a slightly higher chance of retrieval (step 2 in the models' strategies). However, iconic memory also influences the retrieval process through spreading activation. Color values have the highest acuity, hence a higher chance of entering into iconic memory. More color values in iconic memory spread more activation to respective values in declarative memory. As a result, color values have a higher chance of being retrieved from declarative memory and used in dimension-reduction. In picture trials, this process explains why subjects often prefer color for dimension-reduction to any other attribute (Figure 6a). However, in a model for word trials, iconic memory has a negligible influence on the retrieval process, since it is almost empty. Hence, all attributes have a near equal chance of retrieval, thereby removing any possible preference toward a specific attribute.

#### Scanpaths

The model for picture set can prioritize locations for attention shifts to the parts of a scene that are both salient and relevant to the current goal reasonably well. For example, in the trial shown in Figure 11a (and assuming the model is looking for a set among blue cards) it can predict with high accuracy where all of the blue cards are based on the content of iconic memory. Such luxury is not available to the model for word set. Its iconic memory is almost empty, and the model has to shift attention based purely on the prior knowledge of the structure of the scene. It results in a significant difference between picture and word set models in terms of how they scan the search space *G* (steps 4 and 5 in the models' strategies). The picture set model is fairly efficient since it scans only those cards that belong to search space *G*. The word set model cannot identify pre-attentively which cards belong to search space *G*, so it scans all cards. Such scanning is done by shifting attention to the next closest card.

### Model results

Both picture and word set models had to play 100 times through the same block of 32 trials that the subjects did during the experiment. The following sections discuss the results of these runs.

#### Model fit

It is extremely hard to properly estimate a general fit of a model simulating a task as complex as SET. If the models of picture and word set, respectively, are valid, they should produce fixation sequences similar to sequences of human subjects. We have compared subjects' collapsed fixation sequences to sequences produced by models. The resulting comparison scores were taken as an estimation of the models' fits. 

We have used *ScanMatch* [34] as a method for comparing fixation sequences. *ScanMatch* provides several mechanisms that make it more suitable for comparing eye movement data than more conventional methods, such as an estimation of the Levenshtein distance [35]. *ScanMatch* is based on the Needleman–Wunsch algorithm [36] that uses a substitution matrix to maximize the similarity score resulting from a comparison of two sequences. That substitution matrix contains scores for aligning every possible combination of two elements. Comparisons based on that substitution matrix allow for alignments based on overall similarity patterns rather than the binary equalities of individual elements in the sequence. This feature is important considering a certain degree of randomness in the pattern of fixations that arises when a scene is relatively complex.

We created substitution matrices for each trial. Each matrix contained scores for aligning a trial's cards with one another. Scores were calculated based on the similarity of two cards with respect to the highlighted card in the trial. Next, a subject's fixation sequence for each trial was compared to the corresponding 100 fixation sequences produced by the model on the same trial. Finally, the overall mean scores were taken for each subject as an estimation of the model's fit to that particular subject's data. The model's general fit to the experimental data was calculated as a grand mean of all of the subjects' scores. The scores were calculated separately for picture and word trials. We also generated random fixation sequences and compared them to the subjects' sequences the same way the models' fixation sequences were compared. This gives chance-based lower boundaries for similarity scores against which the models' scores can be compared.

Table 4 shows grand means of similarity scores calculated for the models' sequences and random fixation sequences. The fixation sequences produced by the two models have significantly higher similarity scores than the fixation sequences generated randomly. The significance was calculated separately for the two trial types using one-way within-subject ANOVAa. The analysis result indicates that the similarity of the models' fixation sequences to the subjects' sequences is significantly above chance level. We can conclude that both models have an explanatory capability and capture the subjects' behavior at least in some degree. 

**Table 4:** Grand means of similarity scores after comparing each subject's collapsed fixation sequences to randomly and model generated fixation sequences.

	Model	Random	*F(1, 17)*	*p* value
Picture set	*M*=-0.347, *SE*=0.013	*M*=-0.438, *SE*=0.008	219.5	< 0.0001
Word set	*M*=-0.386, *SE*=0.007	*M*=-0.460, *SE*=0.006	359.6	< 0.0001

Finally, we did a cross comparison of fixation sequences between human subjects. The resulting grand means are *M*=-0.317 (SE=0.010) and *M*=-0.294 (SE=0.007) for picture and word trials respectively. Those scores are the upper boundaries of similarity against which the models' fits can be evaluated. Both models definitely do not produce the best possible fit. However, some deviation is expected, considering the quite complex nature of the task. 

#### Reaction times

**Figure 12:**
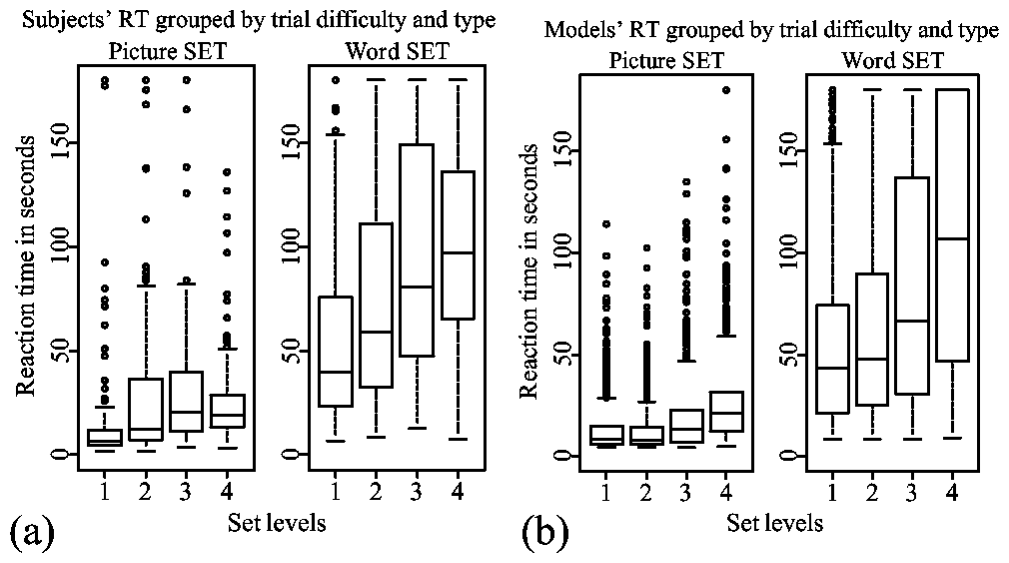
Comparison of (a) subjects' and (b) models' reaction times. Models' reaction times were calculated from 100 runs of picture and word set experiments.

Figure 12 shows boxplots of reaction times for both picture and word set models compared to the respective reaction times from subjects. Both models' reaction times increase as a function of set level. This is to be expected, since at the beginning stages of the trial, both models prefer to search for a set among cards that are similar to a highlighted card. This is essentially a search through dimension-reduction, since the models ensure that cards share at least one attribute value with a highlighted card.

#### Dimension-reduction

**Figure 13:**
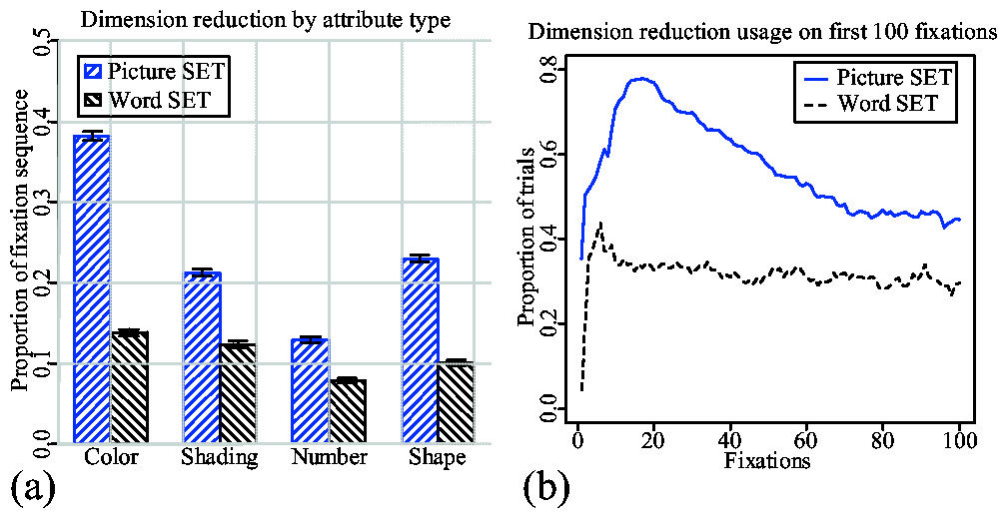
Models' overall usage of dimension reduction in two trial types. (a) The usage of attribute types in similarity-based scanning as a proportion of the trial's fixations sequence. (b) The changing proportion of trials in which dimension-reduction was used. The proportions are calculated as a function of the fixation position within a trial. The proportion on fixation *x* is calculated by counting the trials that have a dimension-reduction block that include fixation *x*. The lengths of blocks from word trials are also normalized to match the length scale of picture trials.

The model for picture set has a relatively high tendency for dimension-reduction, as is shown in Figure 13. Color is prioritized for dimension-reduction more than any other attribute type (Figure 13a), which is similar to our experimental results (Figure 6a). This priority is a result of color values being more readily available in iconic memory than values of any other attribute type due to their higher acuity. Dimension-reduction is preferred at the beginning of the game (Figure 13b) with its usage gradually decreasing as the trial progresses. At step 2 of the model's strategy, the values of the highlighted card have a higher chance of retrieval. However, those same values get inhibited on consecutive retrievals. This simple process results in an overall pattern of dimension-reduction that resembles the one shown by human subjects (Figure 6b). As in our previous study [8], the picture set model exhibits a higher tendency for doing dimension-reduction than the human subjects. The most likely explanation for this difference is that not all instances of dimension-reduction were captured from the human data. Because only blocks of consecutive fixations with a chance probability of less than 5% have been included in the analysis, occasional wandering fixations produced by human subjects can significantly decrease the calculated proportions of dimension-reduction in the human data. On the other hand, the model's attention shifts are precise with no wandering fixations or other forms of noise artifacts.

Figure 13 and Figure 6 show that the model for word set is able to replicate subject behavior even better than the picture set model. The model exhibits a very slow, but steady decrease in dimension-reduction usage over the course of a trial, similar to experimental results (Figure 13b). We did the same mixed-effect linear regression analysis that was done on the experimental data (which includes all fixations in positions between 20 and 80). The main and interaction effects shown in Table 5 indicate that the decrease in dimension reduction in word trials is significant. The reader can also refer to materials S2 for an additional analysis based on ARIMA models applied to data on Figure 13b.

**Table 5:** The results of linear mixed-effect regression analysis of a predicted proportion of dimension reduction based on a fixation position and a trial type.

	Estimate	Std. Error	*t* value	*p* value
Intercept (Picture trial)	0.8399	0.0096	87.49	< 0.001
Fixation position	-0.0053	0.0001	-55.42	< 0.001
Word trial	-0.4898	0.0071	-68.83	< 0.001
Fixation position and word trial interaction	0.0039	0.0001	28.70	< 0.001

As Figure 13a shows, there is no clear preference toward a specific attribute in word trials. This is because the acuity difference among attribute types is gone. However, we know that the word set model uses dimension-reduction in a similar manner as the other model. The obvious question is why there is no clear indication of its usage in Figure 13b. The answer lies in the different scanpaths that the model for word set produces. The paths with dimension-reduction are revealed by identifying subsequences of continuous fixations on cards that share a common value with a highlighted card. It is quite easy to identify such subsequences in scanpaths produced from picture trials, since the model does targeted searches supported by the content of iconic memory. On the other hand, the word set model does exhaustive searches by attending every card. This makes it hard to identify subsequences of continuous fixations on similar cards. This results in the rather uninformative near flat line shown in Figure 13b. The fact that the true proportion of dimension-reduction in word set is as high as 40% becomes apparent in a verification phase. During this verification phase, both subjects and model make consecutive fixations on the same set of cards to verify whether a valid pair was made or the valid set was found. Examples of fixations belonging to the verification phase can be seen in Figure 9, in which such fixations are marked by red and green blocks within the fixation sequence diagram.

**Figure 14:**
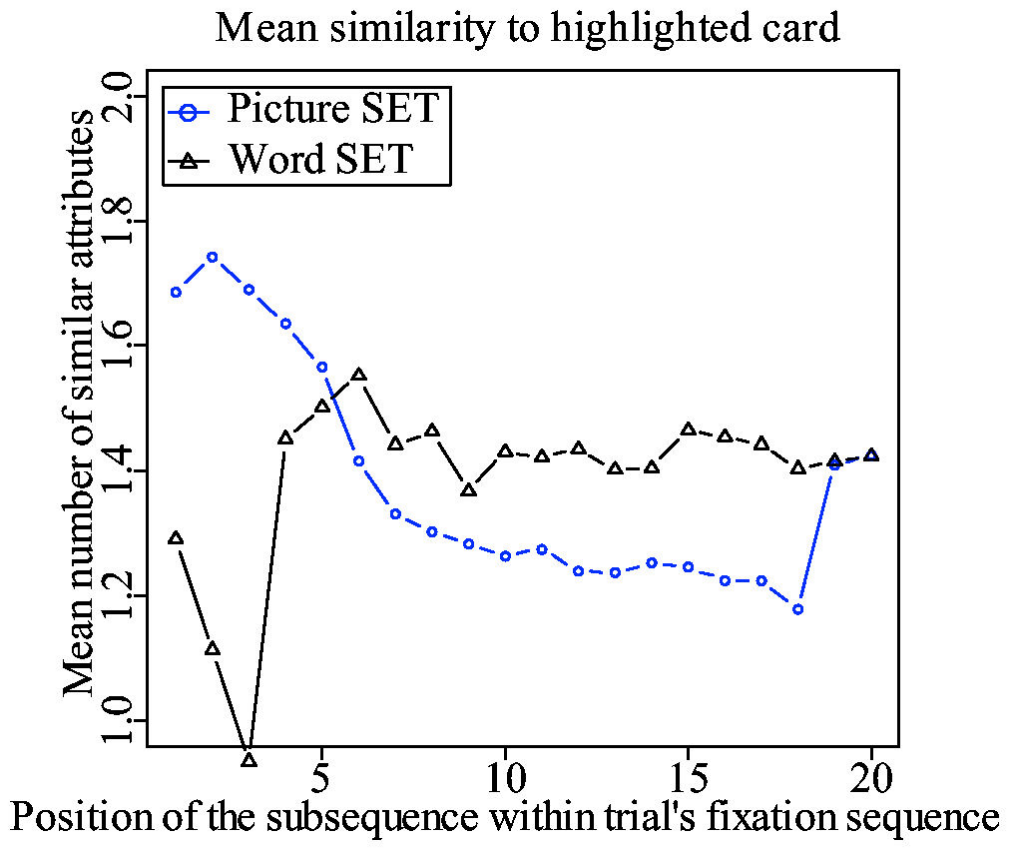
The mean overall similarity of all cards in a particular subsequence to the highlighted card. The values are calculated separately for picture and word trials.

The picture set model shows a clear gradual shift from similarity to dissimilarity-based search. This gradual shift shown in Figure 14 resembles quite closely the one shown in Figure 7 obtained from experimental data. The picture is different for the word set model. The mean similarity to a highlighted card stays on more or less the same level. According to the results of the mixed-effect regression analysis shown on Table 6, there is no decrease in similarity to a highlighted card in word trials. The slight upward bump between the 4th and 9th subsequences is the only visible clue that there is a preference toward similarity at the beginning. This lack of an obvious effect is explained by the same need for an exhaustive search that makes it hard to distinguish dimension-reduction scanpaths from scanpaths where dissimilarity-based search is used.

**Table 6:** The result of a linear mixed-effect regression analysis of a predicted similarity to a highlighted card based on a subsequence's position and a trial type.

	Estimate	Std. Error	*t* value	*p* value
Intercept (Picture trial)	1.585	0.005	324.6	< 0.001
Subsequence position	-0.013	0.000	-20.2	< 0.001
Word trial	-0.197	0.005	-36.6	< 0.001
Subsequence position and word trial interaction	0.014	0.000	18.2	< 0.001

#### Systematic versus unsystematic scanpaths

Similar to human subjects, the two models also show a difference in scanpaths in terms of saccade directions.

**Figure 15:**
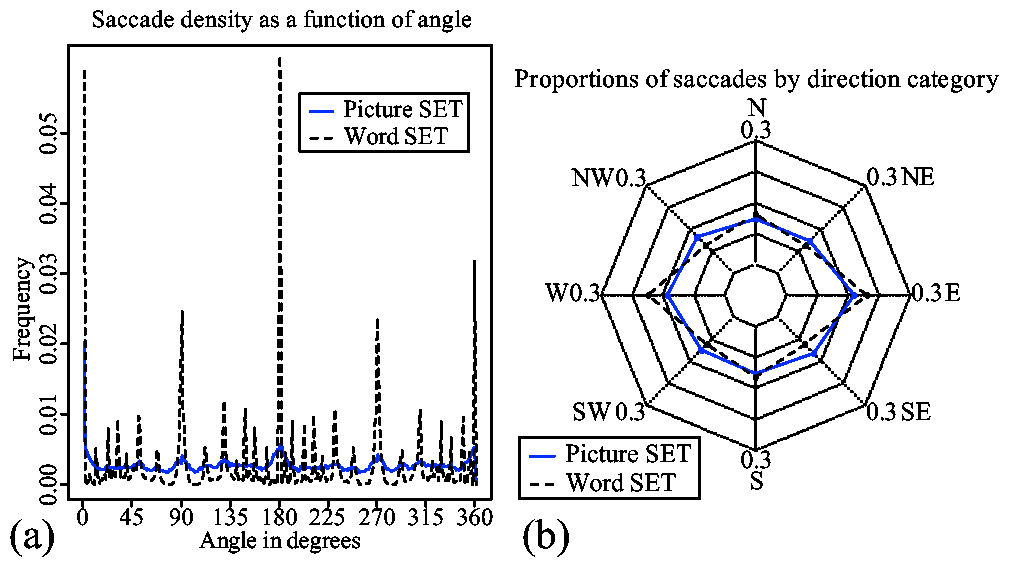
A radar chart for proportions of saccades in each saccade category.

The density plot in Figure 15a clearly shows these differences. Like human subjects, the model for word set shows a higher preference for vertical and horizontal saccades. However, the distributions are narrower and have higher peaks. This is to be expected, since the model is much more precise than human eye movement data.

The radar chart shown in Figure 15b fails to show considerable differences between the two models due to the combined effect of discrete categorization and averaging. However, a logistic mixed-effect regression analysis applied to the model data, shown in Table 7, reveals similar main and interaction effects of trial type and trial level as found in subject data. Probabilities of diagonal saccades in picture and word trials are 0.45 (the intercept) and 0.39 (the main effect of word trial), respectively. Trial level again has a positive effect on the probability of a diagonal saccade in picture trials (the main effect of Trial level). However, such an effect is absent in word trials, as shown by the interaction effect on Table 7.

**Table 7:** The results of logistic mixed-effect regression in which the predicted value is the probability of a diagonal saccade.

	Estimate	Std. Error	*z* value	*p* value
Intercept (Picture trial)	-0.197	0.012	-16.22	< 0.001
Word trial	-0.229	0.013	-17.04	< 0.001
Trial level	0.037	0.004	9.49	< 0.001
Word trial and trial level interaction	-0.041	0.004	-9.33	< 0.001

This decrease in diagonal saccades is mostly the result of an absence of iconic memory content, which would direct attention shifts straight to the cards relevant to the search. Instead, the word set model resorts to shifting attention to the closest card. In most cases, the closest card is a card that is in either in the same column or in the same row.

Figure 15 shows that both models have a tendency toward horizontal saccades with East and *West* having the highest proportions. It is a good fit considering the fact that neither model has an explicit preference for any saccade direction. Another interesting aspect is the fact that both models produce a higher proportion of diagonal saccades than subjects. This should be considered together with the fact that neither of the models do unsystematic searches. At any time, the models' attention shifts are always guided by some top-down goal. This result indicates that an estimation of diagonal/non-diagonal saccades is not the reflection of the systematicity of the search, but rather an indication of the structure and presentation style of the scene.

## General Discussion and Conclusion

In the previous section, we have described two nearly identical models. Both models use exactly the same strategy and the same set of values for adjustable parameters. However, the two models produce behavioral data that on the surface look very different. The entire difference in behavior can be explained by a simple change in presentation style of the task. Furthermore, both models show a good fit to the experimental data, suggesting that a similar change in presentation style affected human subjects in much the same way: the behavioral data may change significantly without changes in overall strategy. 

The contrast between the picture and word versions of SET shows that the style of presentation alone can have a drastic effect on performance in a problem-solving task. With no changes in isomorphic structure, a simple replacement of an iconic representation with a textual representation resulted in more than a twofold increase in reaction times. However, as experimental results and model simulations show, the overall strategy, the way the problem-solving task is approached, did not change. Our original model for picture set was adapted to play word set with the minimum changes necessary to compensate for the absence of the perceptual components of the game. Yet, the model for word set was able to closely replicate subjects' behavior with respect to reaction times and eye movements. Furthermore, the model provides a perspective from a level of individual cognitive processes. Exploring how these processes change based on the nature of a task, helps us to understand how subjects manifest different behaviors in two versions of SET, while still following the same strategy. 

It is interesting how a simple change in visual presentation style can result in what can be called a cascading domino effect in cognitive processes. Change in presentation style triggers change in a cognitive process that itself triggers change in one or more other processes. The changes propagate like a chain reaction. In SET, replacing a high acuity stimulus with a low acuity stimulus removed the advantage of peripheral vision. This lack of peripheral vision resulted in a lack of content in iconic memory and imposed changes on how a visual stimulus, such as a card, was encoded. Changes in iconic memory and encoding further affected the prioritization of attention shifts that manifested itself in different scanpaths. All those changes added up, resulting in increased reaction times and a different pattern of fixation sequences. Furthermore, the data initially appear to give an overall false impression that there are fundamental changes in the strategy subjects use to find a set. However, results of this study do in fact show that changes in presentation style do not necessarily trigger changes in how a subject approaches a problem-solving task. Instead, there are more subtle changes on the level of cognitive processes. The strategy remains the same, but the cognitive processes that are used to implement the overall strategy can change. Such a change can be either beneficial or damaging to performance. For example, in picture trials, peripheral vision is extremely useful in locating cards relevant to the search. In word trials, peripheral vision does not provide any leverage, given that the only option is that of deliberate top-down scanning. This transition from faster low-level processes to more top-down cognition has a rather significant negative effect on reaction times.

Jacob and Hochstein [10], who originally proposed dimension-reduction, assumed that the bias toward similarity in SET is a result of the highly perceptual nature of the game. They argued that players prefer to search for lower level sets, because it is easier to identify similar cards using bottom-up visual processes. Our experiment with word set showed that this is not the case. Even in absence of bottom-up encouragement, subjects needed less time to find lower level sets, indicating that bias toward similarity still exists. This bias is definitely part of a deliberate strategy, rather than an artifact of mechanisms based on perceptual similarity. However, we are yet to identify what exactly causes players to look for similar cards first, rather than for dissimilar cards.

### Exploring beyond SET

It is completely possible that the changes in underlying cognitive processes are responsible for better performances in the Marble Drop game. Meijering at al. [3] also acknowledge the importance of context, although from a perspective of higher-order reasoning. The advantage of Marble Drop is that it provides a bottom-up visual context using colors, trapdoors and bins of decreasing heights. This context is more intuitive and easier to process using bottom-up cognition. One obvious example is the clear advantage peripheral vision provides in Marble Drop. It is much easier to detect difference in color and color-grades using peripheral vision, than to deliberately compare numeric values. It is the possibility to use visual processes that are bottom-up, pre-attentive and parallel that makes Marble Drop an easier game.

### Smarter than expected?

One can argue that bottom-up processes should be able to extract at least some semantic information in order to provide a necessary performance boost in a problem-solving task. 

For example, in Marble Drop, it is easier to visually differentiate and compare payoffs due to a distinct color-grade associated with each payoff. However, this also implies the presence of some form of a semantic association between darker color and a higher payoff at the pre-attentive level. If there is no such association, deliberate comparison will still be necessary. However, there is a mounting amount of research suggesting that pre-attentive visual processes are not as dumb as they were considered to be before [37]. It is often ignored how much information is processed subconsciously [38]. proposed an architecture where a certain amount of semantic information is processed pre-attentively by the human vision system. Perhaps it is exactly that kind of visual information that is readily available in picture trials that makes the original version of the game so much easier than the word version of the game. In word trials, the semantic information that otherwise would have been extracted more efficiently by visual bottom-up processes needs to be processed by deliberate top-down reasoning.

### Exploring through models

Models are useful tools for exploring differences that are otherwise difficult to reveal by means of statistical analysis. Computer modeling is the only objective way currently available to explore the behavior of a complex modular system in which changes in one module can propagate throughout the entire system. The human cognitive system is definitely a good representative of such. For example, it is hard to statistically calculate the outcomes of the domino effect described in the previous subsection. Instead, we used a computational model based on a cognitive architecture to directly simulate these outcomes. The model for word set worked quite well, especially considering the fact that it was directly adapted from the existing model of picture set with minimal changes to suit the new presentation style.

### Data and source code

All of the data related to this study, including the model source code and the experimental data can be downloaded via the following link: http://www.ai.rug.nl/~n_egii/models/. The source code for the PAAV module can be downloaded at http://www.ai.rug.nl/~n_egii/models/codes/paav-module-no-vstm.lisp. The source code for ACT-R architecture and the modules of Threaded Cognition and Base-Level Inhibition are freely accessible at http://act-r.psy.cmu.edu/.

## Supporting Information

materials S1 **Results from analysis of residuals of ARIMA models applied to proportions of dimension reduction usage by human subjects.**
(DOC)

materials S2 **Results from analysis of residuals of ARIMA models applied to proportions of dimension reduction usage by ACT-R models.**
(DOC)
