# Supplementary material for: The Effect of Visual Representation Style in Problem-Solving: A Perspective from Cognitive Processes
Source: PLoS One. 2013 Nov 15;8(11):e80550. doi: 10.1371/journal.pone.0080550 (PMC3829871; doi:10.1371/journal.pone.0080550)
Supplement: materials S1 — Results from analysis of residuals of ARIMA models applied to proportions of dimension reduction usage by human subjects. (DOC) [file pone.0080550.s001.doc]

**Supplementary Material S1: Results from analysis of residuals of ARIMA models applied to proportions of dimension reduction usage by human subjects**

We used ARIMA models to reveal the trends in dimension reduction data shown in Figure 6b. In both word and picture set, we used points from the first 70 fixations as time series for building the ARIMA models. Both ACF and PACF were non-significant for both time series after the first order differencing (figures S1-1b and S1-2b). Therefore, we used ARIMA of order (1, 0, 1) with no seasonal component and no constant terms included in the models. In ARIMA model for picture set, the AR(1) coefficient is 0.9953 (*SE = 0.0053, |z| = 187.7925*), and MA(1) coefficient is 0.2047 (SE = 0.1106, *|z| = 1.851*). In ARIMA model for word set ARIMA model, AR(1) coefficient is 0.9978 (*SE = 0.0028, |z| = 356.3571*) and MA(1) coefficient is 0.0354 (*SE = 0.1099, |z| = 0.322111*). All coefficients, except MA(1) coefficient in word set model, are significant. We did a residual diagnostics as goodness-of-fit tests. In both models, the residuals show no trend with no significant correlations present among ACF values of residuals. The Ljung-Box-Pierce statistics done for each lag up to 20 resulted in all non-significant p-values for both ARIMA models (figures S1-3 and S1-4). Overall, fit for both models were good. We used both models to forecast future trends of using dimension reduction for 60 fixations ahead. The forecast are shown in Figure S1-5. Forecasts show downward trends in usage of dimension reduction usage that is in conformance with results from previous analysis.

**Figure S1-1. ACF and PACF graphs of proportions of dimension reduction usage for a picture trial (a) with no differencing applied and (b) with the first order differencing.**

(a)

(b)

**Figure S1-2. ACF and PACF graphs of proportions of dimension reduction usage for a word trial (a) with no differencing applied and (b) with the first order differencing.**

(a)

(b)

**Figure S1-3. Analysis of residuals for testing a goodness-of-fit of ARIMA(1, 0, 1) model to picture set data.**

**Figure S1-4. Analysis of residuals for testing a goodness-of-fit of ARIMA(1, 0, 1) model to word set data.**

**Figure S1-5. The proportions of dimension reduction usage in word and picture trials as predicted by ARIMA models.**
