# Supplementary material for: The Effect of Visual Representation Style in Problem-Solving: A Perspective from Cognitive Processes
Source: PLoS One. 2013 Nov 15;8(11):e80550. doi: 10.1371/journal.pone.0080550 (PMC3829871; doi:10.1371/journal.pone.0080550)
Supplement: materials S2 — Results from analysis of residuals of ARIMA models applied to proportions of dimension reduction usage by ACT-R models. (DOC) [file pone.0080550.s002.doc]

**Supplementary Material S2: Results from analysis of residuals of ARIMA models applied to proportions of dimension reduction usage by ACT-R models**

We also used ARIMA models to reveal the trends in models' dimension reduction data. Similar to analysis of human data, we used points from the first 70 fixations as representatives of the time series. Both ACF and PACF were non-significant for picture set time series after the first order differencing (Figure S2-1b). Both ACF and PACF decayed quickly for word set time series without any differencing (Figure S2-2a). Therefore, we used no differencing in both ARIMA models. No seasonal component and no constant terms were included in the models. A square root transformation was done on the picture set data for a better fit of the ARIMA model. We used models with (2, 0, 1) and (1, 0, 1) orders for picture and word sets respectively. Table S2-1 shows the resulting fitted coefficients. Absolute *z* values indicate that all coefficients are significant.

**Table S2-1. Fitted coefficients for ARIMA(2, 0, 1) and ARIMA(1, 0, 1) models for picture and word set respectively.**

|  | Picture set | | | Word set | |
| --- | --- | --- | --- | --- | --- |
|  | AR(1) | AR(2) | MA(1) | AR(1) | MA(1) |
| Coefficients | 1.9938 | -0.9946 | -0.7787 | 0.9876 | 0.5452 |
| S.E. | 0.0095 | 0.0096 | 0.0990 | 0.0119 | 0.0937 |
| |z| | 209.87 | 103.60 | 7.87 | 82.99 | 5.82 |

We did a residual diagnostics as goodness-of-fit tests. In both models, the residuals show no trend with no significant correlations present among ACF values of residuals. The Ljung-Box-Pierce statistics done for each lag up to 20 resulted in all non-significant p-values for both ARIMA models (figures S2-3 and S2-4). Overall, models fit well the data and can be used for predictions. We used both models to forecast future trends of using dimension reduction for 60 fixations ahead. The forecast are shown in Figure S2-5. Similar to human data, forecasts show downward trends in usage of dimension reduction usage.

**Figure S2-1: ACF and PACF graphs of proportions of dimension reduction usage for a picture trial (a) with no differencing applied and (b) with the first order differencing.**

(a)

(b)

**Figure S2-2: ACF and PACF graphs of proportions of dimension reduction usage for a word trial (a) with no differencing applied and (b) with the first order differencing.**

(a)

(b)

**Figure S2-3: Analysis of residuals for testing a goodness-of-fit of ARIMA(2, 0, 1) model of picture set data.**

**Figure S2-4: Analysis of residuals for testing a goodness-of-fit of ARIMA(1, 0, 1) model of word set data.**

**Figure S2-5. Models' proportions of dimension reduction usage in word and picture trials as predicted by ARIMA models.**
